# Screening of FDA-Approved Small Molecules to Discover Inhibitors of the *Pseudomonas aeruginosa* Quorum-Sensing Enzyme, PqsE

**DOI:** 10.1021/acs.biochem.5c00475

**Published:** 2026-01-14

**Authors:** Hannah A. Jones, Mary J. Baxter, Nicolas Zimmermann, Ada Li, Katelynn A. Perrault Uptmor, Isabelle R. Taylor

**Affiliations:** Department of Chemistry, 8604 William & Mary, Williamsburg, Virginia 23185, United States

## Abstract

*Pseudomonas aeruginosa* is a notorious pathogen that is a leading cause of hospital-acquired infections, for which there are few treatment options. The quorum sensing (QS) pathway governs many pathogenic behaviors that allow for *P*. *aeruginosa* to stage infections. Within the QS pathway, there is a key protein–protein interaction between an enzyme, PqsE, and one of the master QS regulators, RhlR. Although its catalytic function is dispensable for its interaction with RhlR, previous mutagenic work characterizing the active site of PqsE identified active site mutations that induce a conformational change in PqsE, preventing it from forming a complex with RhlR. These active site mutations, when introduced stably into the genome of *P*. *aeruginosa*, also lead to a significant decrease in production of a key toxin, pyocyanin, and prevent colonization in the lungs of a murine host. Here, we performed a fluorescence polarization screen of an FDA-approved drug library to identify molecules that bind in the active site of PqsE. Three molecules were identified, two of which showed inhibitory activity consistent with a competitive mode of inhibition. One hit molecule, Apomorphine, had a distinctly different inhibitory profile and is potentially binding outside of the active site to allosterically inhibit enzyme activity of PqsE. All three hit molecules were tested in a cellular enzyme assay, and one of the competitive inhibitors, Vorinostat, was found to inhibit intracellular PqsE. Vorinostat is now being explored as a candidate for synthetic derivatization to inhibit the PqsE-RhlR protein–protein interaction via binding in the PqsE active site.

## Graphical abstract

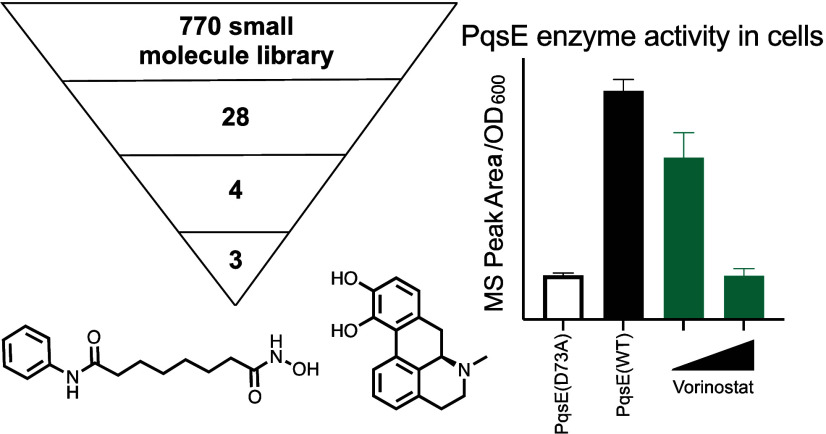

The human pathogen, *Pseudomonas aeruginosa*, is a leading cause of nosocomial infections and a significant burden to human health.^1,2^ In addition to the many virulence factors produced by *P*. *aeruginosa*, the species is capable of carrying out coordinated group behaviors such as biofilm formation that contribute to its heightened pathogenicity.^3^ These behaviors are largely under the control of the bacterial cell-to-cell communication system called quorum sensing (QS). Through the production, release, and detection of small molecule signals, called autoinducers, *P*. *aeruginosa* uses QS in order to make a coordinated lifestyle switch between carrying out single-cell and group behaviors.^4,5^ This lifestyle switch, which is marked by widespread changes in gene expression, occurs once the concentration of autoinducers in the surrounding environment reaches a critical threshold, signifying that the local population has reached high-cell density. Activation of the QS system at high-cell density is critical for the ability of *P*. *aeruginosa* to stage an infection in a host, and therefore, it is of great interest as a target system for the development of new antimicrobial agents.^6^

There are three main systems that make up the QS network in *P*. *aeruginosa*, two of which (the Las and Rhl systems) consist of a LuxI/R autoinducer synthase/receptor pair and one (the Pqs system), more specialized to *Pseudomonads*, that consists of a LysR receptor and a biosynthetic operon dedicated to the production of its respective autoinducer.^7^ The Las system consists of a synthase, LasI, responsible for producing the autoinducer N-(3-oxo-dodecanoyl)-L-homoserine lactone (3-oxo-C12-HSL), and the receptor that binds 3-oxo-C12-HSL, LasR.^8^ For the Rhl system, RhlI is the synthase that produces the autoinducer, N-butanoyl-L-homoserine lactone (C4-HSL), which binds the receptor, RhlR.^9^ These receptors, when bound to their respective autoinducers, are activated as transcription factors that control the expression of hundreds of genes involved in group behaviors.^10^ For the Pqs system, the enzymes PqsA-D along with PqsH are collectively responsible for the synthesis of the *Pseudomonas* Quinolone Signal (PQS)^11^ which binds and activates the receptor PqsR. These three systems are highly interconnected, and the regulons of LasR, RhlR, and PqsR consist of genes regulated by all three, two, or only one of the receptors.^12–14^ As an added layer of connectivity, the fifth gene of the *pqsABCDE* operon is dispensable for the production of PQS^11^ but indispensable for the activation of a large portion of the RhlR regulon.^15–17^ This is due to a nonenzymatic function of the PqsE protein, which is its ability to form a complex with RhlR.^18–20^

The PqsE-RhlR protein–protein interaction is crucial for the ability of *P*. *aeruginosa* to carry out a range of behaviors, including the production of a toxin, pyocyanin.^21,22^ Interestingly, mutations introduced into the PqsE active site have been shown to cause a conformational shift that disrupts the interaction with RhlR at a distal site. Specifically, the substitution of an active site glutamate with tryptophan (E182W) causes a loop rearrangement which drastically weakens the affinity of PqsE for RhlR and almost entirely inhibits pyocyanin production.^21,23^ The introduction of the E182W substitution to the PqsE active site was even enough to prevent colonization of *P*. *aeruginosa* in the lungs of a murine host.^23^ For this reason, the PqsE active site is being explored as a target for the development of small molecule modulators, despite the finding that PqsE enzyme activity is dispensable for the PqsE-RhlR interaction.^21^ In this study, we carried out a screen for molecules that compete with an active-site-targeted probe, BB562, to bind PqsE.

The initial stage of screening was performed using a fluorescence polarization (FP) assay testing 770 molecules of the SCREEN-WELL FDA-approved drug library V2 from Enzo Life Science. This screen was designed to test whether the molecules could compete with the previously published BB562 fluorescent probe to bind in the PqsE active site.^21^ Each molecule in the library was tested at a single concentration of 250 μM with 2 μM purified PqsE, and a hit was determined to be any molecule that decreased fluorescence polarization by at least 25% when compared to the no-compound control (Figure 1a). Additional controls measuring fluorescence polarization of the probe alone, in the absence of purified PqsE, were included on every plate. The initial single-dose screen yielded 28 molecules that were able to decrease fluorescence polarization by at least the target margin and moved into the next stage of the screen, a test for dose-dependent competitive binding (Figure 1b).

**Figure 1:**
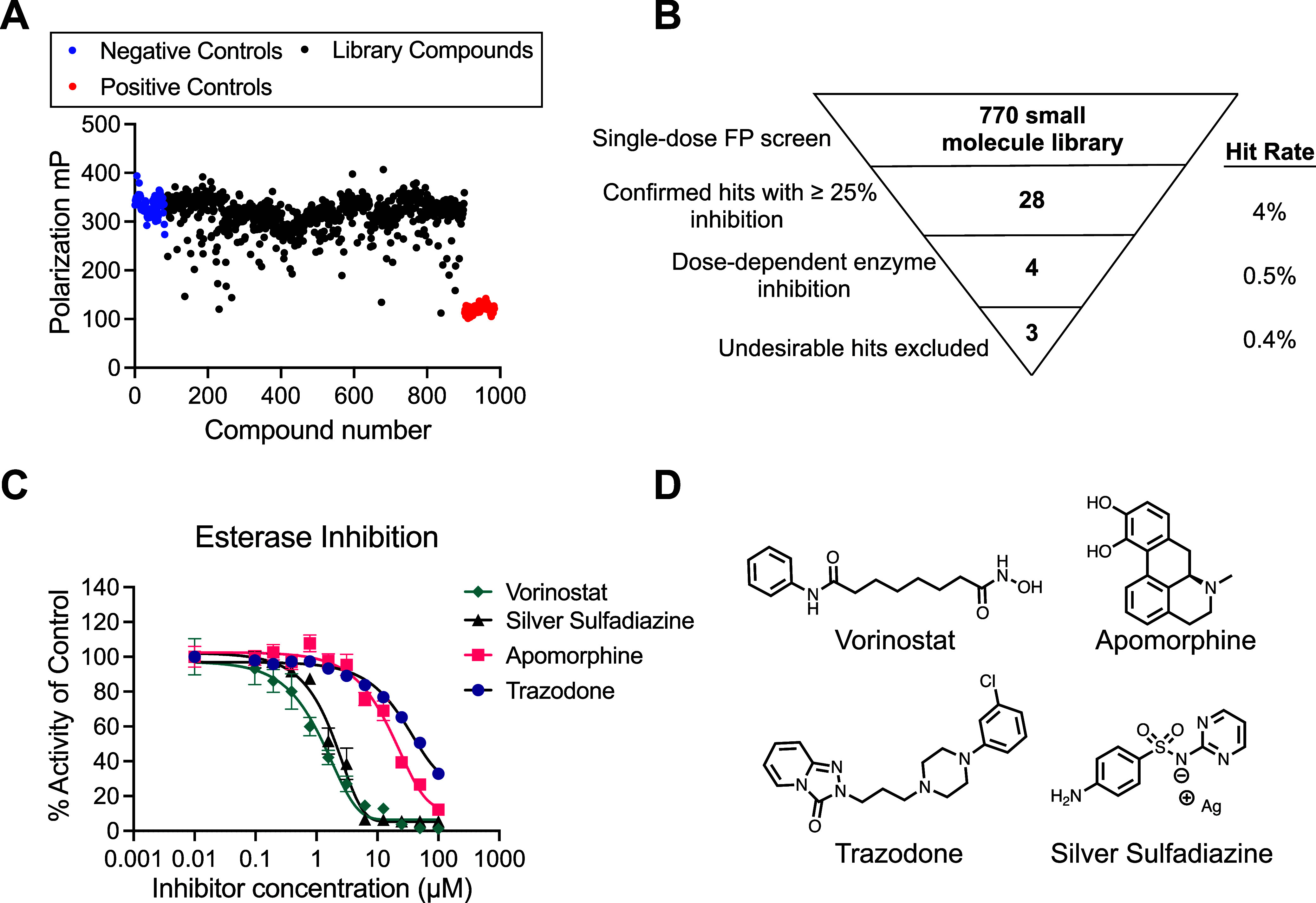
Competitive fluorescence polarization screen of the FDA-approved small molecule library. A) Raw polarization values (in mP) obtained in initial signal-dose screen. Blue points represent the negative control with PqsE treated with DMSO, and red dots represent the positive control containing no protein. All black points represent library molecules and the fluorescence polarization measured for PqsE treated with that compound in the presence of the fluorescent probe, BB562. B) 770 molecules were screened at 250 μM, and 28 molecules were identified to decrease fluorescence polarization by at least 25% compared to the DMSO-treated control wells (hit rate = 4%). Dose–response testing confirmed four molecules as hits, one of which was eliminated from further testing due to suspected off-target activity (hit rate = 0.4%). C) Dose-dependent inhibition of PqsE-catalyzed hydrolysis of MU-butyrate. % Activity is relative to the 0 μM, DMSO-treated protein, and background fluorescence of the inhibitor dilution series in the absence of PqsE was subtracted from the data prior to normalization. Data points represent the average of technical triplicate values, and error bars represent standard deviation. D) Structures of the four confirmed dose-dependent screening hits.

The second stage of the screen took the 28 hit molecules from stage one and tested them in the same fluorescence polarization assay but in a dilution series. At this stage, the molecules were also tested for any potential spectral interference, as fluorescence polarization was measured for the dilution series of each test molecule plus the BB562 probe in the absence of PqsE. True hits were determined to be any molecule that displayed a full inhibition curve (one exception being Trazodone, which did not reduce fluorescence polarization fully to baseline levels) (Figure S1). Of the 28 molecules tested, four dose-dependent hits were identified and purchased for further exploration.

The four dose-dependent hit molecules were then tested for their ability to inhibit an enzymatic activity of PqsE. PqsE is annotated as a thioesterase but has broad hydrolase activity and is able to cleave a wide range of synthetic substrates *in vitro*. The hydrolysis of 4-methylumbelliferyl butyrate (MU-butyrate) has been used previously as a measure of PqsE enzyme activity.^21–23^ The four hit molecules were all able to dose-dependently inhibit PqsE enzyme activity with low- to mid-micromolar potencies (IC_50_ values of 1.2 μM, 1.8 μM, 18 μM, and 180 μM for Vorinostat, Silver Sulfadiazine, Apomorphine, and Trazodone, respectively) (Figure 1c). Upon further testing, Silver Sulfadiazine was eliminated from consideration due to its likely nonspecific binding and destabilization of the protein. While exhibiting potent competition with the BB562 probe and inhibition of PqsE esterase activity, Silver Sulfadiazine was found to kill *P*. *aeruginosa* and, thus, did not lend itself to further mechanistic testing in cells.^24^

The hit molecules Vorinostat, Apomorphine, and Trazodone were analyzed further to understand the mechanism by which they decreased the enzyme function. Silver Sulfadiazine was also tested in this manner (Figure S2), although the molecule was not subjected to further analysis. To this end, a previously established PqsE variant, PqsE­(S285W), was used as a tool to determine whether the hit molecules were in fact directly binding in the active site of PqsE. The PqsE­(S285W) variant has a partially blocked active site while retaining normal enzyme function compared to PqsE­(WT).^23^ Although the hydrolytic capacity of PqsE­(S285W) is similar to that of the WT protein, this protein exhibits an affinity for the BB562 probe compared to PqsE­(WT). If a hit molecule is binding in the active site, we would expect that molecule to have reduced potency against the PqsE­(S285W) variant enzyme. Each of the hit molecules was tested in a multipoint dilution series against both PqsE­(WT) and PqsE­(S285W) in an esterase assay (Figure 2). Interestingly, only two molecules showed a decrease in potency against PqsE­(S285W) (Figure 2a,b). The IC_50_ value of Vorinostat increased by 10-fold against PqsE­(S285W), suggesting that this molecule binds in a way that is affected by the substitution of a serine (short side chain, hydrogen bond donor/acceptor) with a tryptophan (bulky side chain, no hydroxyl moiety for hydrogen bonding). Trazodone was also less potent against the PqsE­(S285W) variant; however, due to low potency and an incomplete inhibition curve, an IC_50_ value could not be determined. Apomorphine did not follow this trend (Figure 2c). Instead, the IC_50_ of Apomorphine was relatively unchanged for the PqsE­(S285W) variant compared to PqsE­(WT) (22 vs 26 μM, respectively). This suggests Apomorphine could be binding outside of the active site and allosterically affecting both binding of the BB562 probe and enzyme activity. This result is consistent with the structure of Apomorphine, which is likely too bulky to bind in the active site of PqsE (Figure 1d).

**Figure 2:**
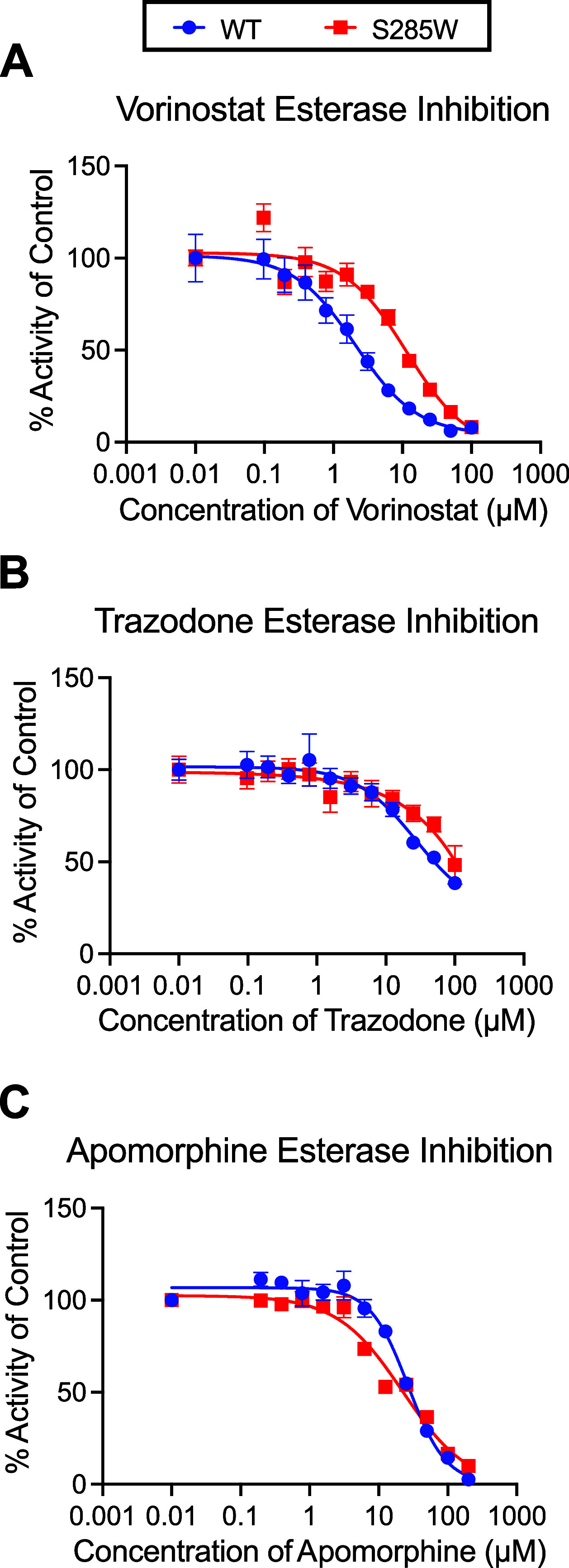
Inhibition of PqsE­(WT) and PqsE­(S285W) by screening hits. Esterase activity of PqsE­(WT) (blue circles) and PqsE­(S285W) (red squares) treated with varying concentrations of A) Vorinostat, B) Trazodone, and C) Apomorphine. % Activity is relative to the 0 μM, DMSO-treated protein, and background fluorescence of the inhibitor dilution series in the absence of PqsE was subtracted from the data prior to normalization. Data points represent the average of technical triplicate values, and error bars represent standard deviation.

The three hit molecules were also tested in *P*. *aeruginosa* cultures to determine their effects on QS-directed behaviors, such as pyocyanin production. Pyocyanin production is an important virulence-related activity that is highly correlated to the ability of *P*. *aeruginosa* to infect a host.^25–27^ The ability to produce pyocyanin is dependent on the PqsE-RhlR protein–protein interaction. This protein–protein interaction, however, is completely independent of the PqsE enzyme activity. Therefore, a molecule would only be able to inhibit pyocyanin production through a PqsE-dependent mechanism if that molecule is inhibiting the ability of PqsE to interact with RhlR. We constructed an assay to determine whether any inhibition of pyocyanin production was specifically achieved via a PqsE-dependent mechanism. This was accomplished following the creation of a *P*. *aeruginosa* strain with an *azeB-lux* reporter.^28^ This reporter strain produces bioluminescence when the *azeB* gene is activated. In *P*. *aeruginosa*, the *azeB* gene is regulated by RhlR. However, unlike most RhlR-regulated genes, the interaction of PqsE with RhlR decreases expression of *azeB*.^28^ In this reporter strain, if the PqsE-RhlR complex is inhibited by a small molecule, we would expect to see a dose-dependent decrease in pyocyanin production as well as an increase in luminescence, meaning increased expression of *azeB*.

The three hit molecules, Vorinostat, Trazodone, and Apomorphine, were tested in this dual assay to look for on-target QS inhibition (Figure 3). Each molecule was tested at 50 and 100 μM in order to look for a dose-dependent trend. Each molecule was tested against two strains of *P*. *aeruginosa*: one that expressed *pqsE­(WT)* and a Δ*pqsE* strain. This way, if any molecules had nonspecific effects on luminescence (i.e., via inhibition of luciferase), this could be determined. All three molecules showed some slight effects on pyocyanin production, although none were statistically significant, and mostly these effects were not dose-dependent. More importantly, the decreases in pyocyanin production were not accompanied by increases in *azeB* expression, suggesting that the mechanism by which pyocyanin production is being inhibited is not dependent on PqsE. This finding was supported by an *in vitro* pull-down assay, in which all three compounds failed to inhibit complex formation between PqsE and RhlR (Figure S3). Interestingly, an increase in absorbance at 695 nm was observed in the Δ*pqsE* strain treated with Apomorphine (Figure 3a), but this was likely due to the slightly green color Apomorphine takes on upon oxidation.^29,30^

**Figure 3:**
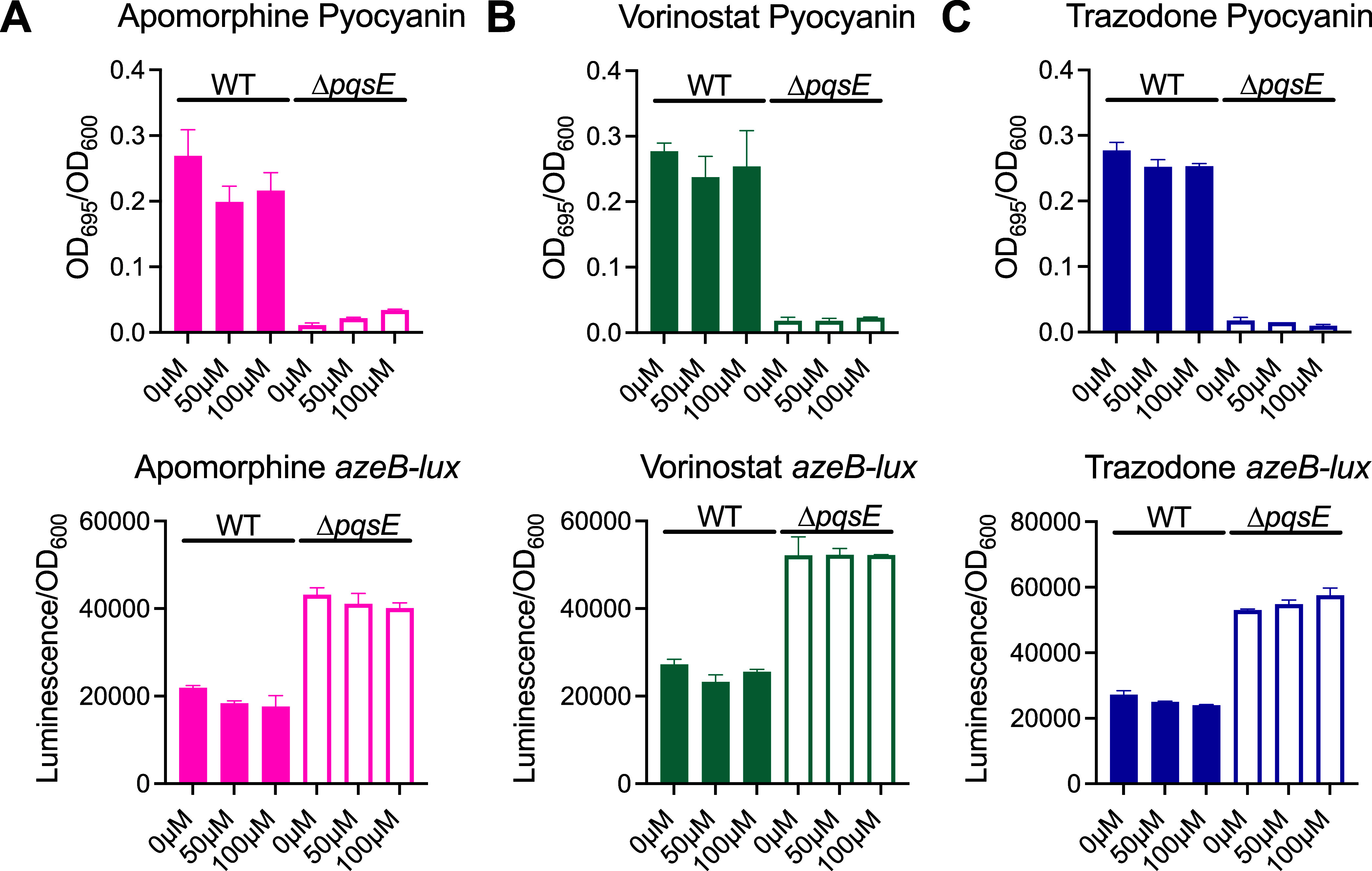
Cellular activity assays for pyocyanin production and *azeB* expression. Pyocyanin production is reported as the OD_695_ of the culture supernatants divided by the OD_600_ of the resuspended pellet. Activation of a transcriptional *azeB-luxCDABE* reporter was measured for the suspended pellet and reported as luminescence divided by the OD_600_. The effect on both pyocyanin production and *azeB* expression was determined for A) Apomorphine, B) Vorinostat, and C) Trazodone in both a strain of *P*. *aeruginosa* expressing *pqsE­(WT)* (filled bars) and *ΔpqsE* (unfilled bars). Graphs show the average of two biological replicates. Error bars represent standard deviation.

The enzymatic function of PqsE is redundant to the biosynthetic pathway dedicated to producing the *Pseudomonas* Quinolone Signal (PQS), as demonstrated by the ability of Δ*pqsE P*. *aeruginosa* PA14 and PAO1 strains to produce PQS to wildtype levels.^18,31^ Alternate thioesterases, such as TesB, have been proposed to fulfill this function in the absence of PqsE enzyme activity.^11^ Although production of the end products of the pathway will not indicate any difference in PqsE enzymatic activity, other intermediates and side-products of the PQS pathway have been scrutinized to measure intracellular PqsE enzyme activity.^11,31^ PqsE performs a thioesterase function within the PQS synthetic pathway in order to convert 2-aminobenzoyl acetyl-CoA to 2-aminobenzoyl acetate (2-ABA) (Figure 4a). 2-ABA is inherently unstable and, to some extent, is decarboxylated to form a side-product, 2-aminoacetophenone (2-AA). 2-AA is a volatile molecule, and the characteristic fruity smell of *P*. *aeruginosa* cultures is attributed to its production.^32^ 2-AA has previously been used as a biomarker for the diagnosis of *P*. *aeruginosa* infections.^33^ We initially looked to 2-AA as a possible analyte for measuring the enzymatic activity of PqsE in *P*. *aeruginosa*; however, when comparing 2-AA levels in the supernatants of a strain harboring catalytically inactive PqsE­(D73A) compared to PqsE­(WT), there was no significant difference in 2-AA levels, as detected by comprehensive two-dimensional gas chromatography coupled with time-of-flight mass spectrometry (GC×GC-TOFMS). Further comparison of all analytes identified in the culture supernatants revealed that there was a statistically significant difference in concentrations of another molecule, acetophenone,^34,35^ between the WT strain and the *pqsE­(D73A)* strain (Figure 4b). We reasoned that this could be a further breakdown product of 2-ABA and therefore chose acetophenone as the analyte for measuring the intracellular enzyme activity of PqsE.

**Figure 4:**
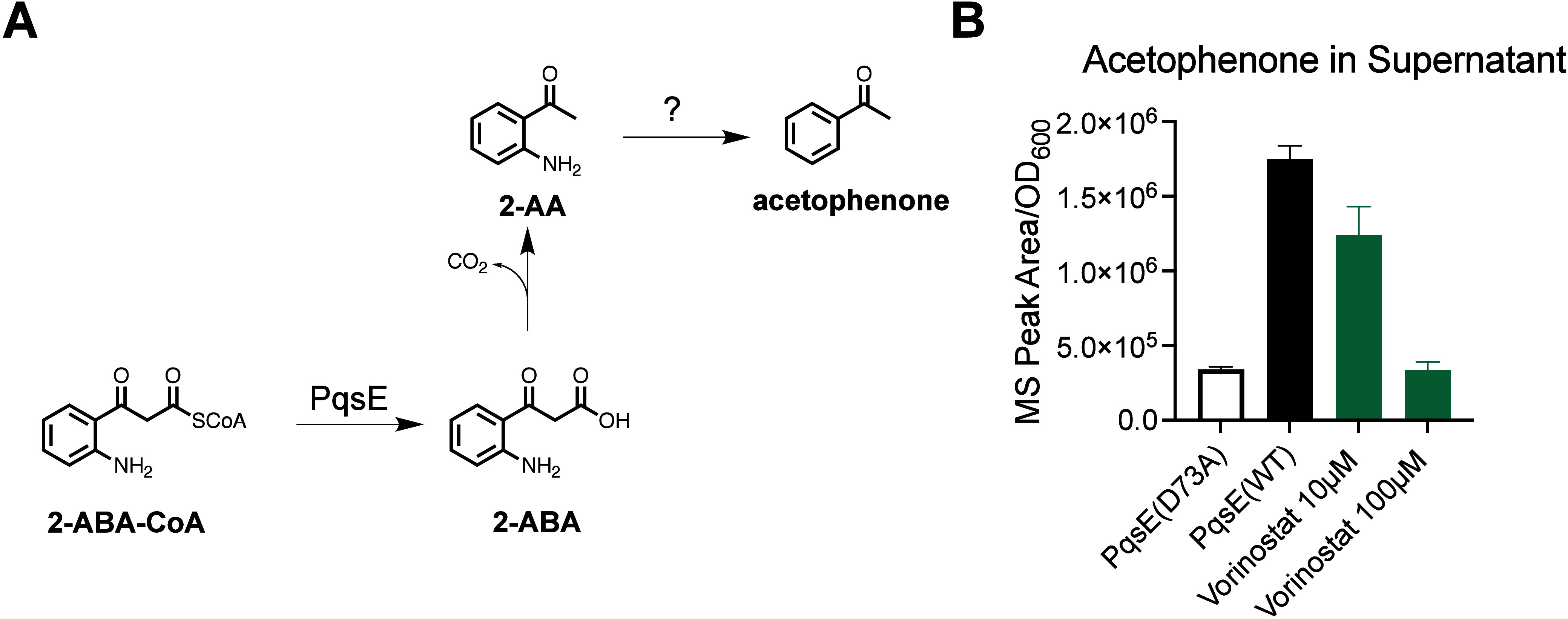
Assay for PqsE enzyme activity in *P*. *aeruginosa*. A) Reaction scheme for annotated PqsE catalytic function in PQS biosynthetic pathway and known byproducts. The mechanism for PqsE-dependent production of acetophenone is uncertain and is therefore annotated with “?”. B) Effect of Vorinostat on acetophenone production. Acetophenone levels detected in culture supernatants from *P*. *aeruginosa* strains grown for 8 h in the presence of specified concentrations of Vorinostat. All MS peak areas were first baseline-subtracted by the peak area detected in uninoculated LB and then normalized to OD_600_ of the suspended cultures to account for any differential growth rates. Data shown are the average of three biological replicates, and error bars represent standard deviation.

To test whether the hit molecules Vorinostat, Apomorphine, and Trazodone inhibit PqsE enzyme activity in *P*. *aeruginosa*, cultures were grown in the presence of the inhibitors at 10 and 100 μM and acetophenone levels were measured in the culture supernatants after 8 h of growth. Compared to the *pqsE­(D73A)* strain, the normalized peak area for acetophenone was approximately 5× greater for wildtype *P*. *aeruginosa*. When treated with 10 μM Vorinostat, there was a 30% decrease in acetophenone, and when exposed to 100 μM Vorinostat, the amount of acetophenone detected dropped to the same level as detected in the *pqsE­(D73A)* strain, suggesting full inhibition of PqsE catalytic activity (Figure 4b). Treating *P*. *aeruginosa* with either Apomorphine or Trazodone had no significant effect on acetophenone levels (Figure S4), suggesting that either these molecules are incapable of binding PqsE intracellularly or they are not potent enough to have a measurable effect on PqsE enzyme activity in cells. Either way, these molecules may require further synthetic optimization for use in *P*. *aeruginosa*.

Due to its heightened antimicrobial resistance and a lack of viable treatment options, there is great interest in exploring new potential mechanisms for antibiotics to treat *P*. *aeruginosa* infections.^36^ Targeting virulence pathways in a way that would disarm but not kill bacteria is a strategy that is gaining traction in recent years, as the theory is, such an anti-infective would be less likely to spur the evolution of resistance mechanisms.^37,38^ Furthermore, there has been an increase in screening of FDA-approved small molecules, as this significantly expedites the drug discovery process.^39,40^ The Enzo FDA-approved molecule library ultimately yielded three molecules to explore in greater depth: Apomorphine, Vorinostat, and Trazodone. The hits were discovered using a multistep screening process to identify molecules that competitively bind in the active site of PqsE. One surprising outcome of this screening strategy was that it not only identified competitive active site binders but also identified one molecule, Apomorphine, that appears to bind outside of the active site and allosterically inhibit PqsE enzyme activity. These three hit molecules are all being explored further to determine their binding modes via structural techniques (primarily crystallography). We are particularly interested in solving a structure of Apomorphine bound to PqsE, as this would potentially be the first structure of a small molecule inhibitor bound to PqsE outside of the active site.

Upon further cell-based analysis, only Vorinostat was found to inhibit PqsE enzyme activity in *P*. *aeruginosa*. This is particularly encouraging as previous screening efforts have produced molecules that inhibit PqsE enzyme activity *in vitro*, but those molecules were found to have no activity in cells due to their probable membrane impermeability^41^ or inhibited PqsE enzyme activity in cells, but were small fragment molecules that exhibited relatively weak inhibition of the purified enzyme.^31^ This suggests that Vorinostat could serve as a starting point for further synthetic diversification and would already possess the ability to cross the *P*. *aeruginosa* membrane and reach its target, PqsE, intracellularly. Although Apomorphine and Trazodone did not appear to inhibit the intracellular enzyme activity of PqsE, these molecules were significantly less potent inhibitors *in vitro*, and their lack of activity in *P*. *aeruginosa* could be due to their low potency.

Apomorphine is still of great interest due to its structural diversity compared to previously identified PqsE inhibitors and potential new mode of action. There is compelling evidence due to Apomorphine’s structure and the PqsE­(S285W) esterase inhibition curve that supports the idea that the molecule is binding to an allosteric site on PqsE. Again, a cocrystal structure of Apomorphine bound to PqsE will reveal more about the mode of action of this inhibitor and whether it represents a viable pathway to explore for PqsE-RhlR complex inhibition. In addition to crystallography, we are exploring alternate ways to measure the ability of small molecules to trap PqsE in a configuration similar to that of PqsE­(E182W). Some of these methods could be scaled to be able to perform new high-throughput screens for such molecules.

## Supplementary Material



## Data Availability

The data supporting this article have been included as part of the Supporting Information.

## Notes

Full dose–response curves for hits from initial single-dose screening, esterase inhibition curves for Silver Sulfadiazine, a gel image for an *in vitro* pull-down assay, MS peak areas and OD_600_ from the cellular enzyme assay, one Table of strains used in the study, and Materials and Methods (PDF)

## Notes

• PqsE: A0A0H2Z6F6 • RhlR: A0A0H2ZEG8
