# Supplementary material for: Screening of FDA-Approved Small Molecules to Discover Inhibitors of the Pseudomonas aeruginosa Quorum-Sensing Enzyme, PqsE
Source: Biochemistry. 2026 Jan 14;65(3):263–9. doi: 10.1021/acs.biochem.5c00475 (PMC12874371; doi:10.1021/acs.biochem.5c00475)
Supplement: Supplementary file 1 [file bi5c00475_si_001.pdf]

Supporting Information for

Screening of FDA-approved small molecules to discover inhibitors of the *Pseudomonas aeruginosa* quorum-sensing enzyme, PqsE

Hannah A. Jones[1], Mary J. Baxter[1], Nicolas Zimmermann[1], Ada Li[1], Katelynn A. Perrault Uptmor[1], Isabelle R. Taylor[1*]

[1]Department of Chemistry, William & Mary, Williamsburg, VA 23185, USA

[*]To whom correspondence should be addressed. Email: irtaylor@wm.edu

This PDF file includes:

Figure S1
Figure S2
Figure S3
Figure S4
Table S1
Materials and Methods

Supplementary Figures

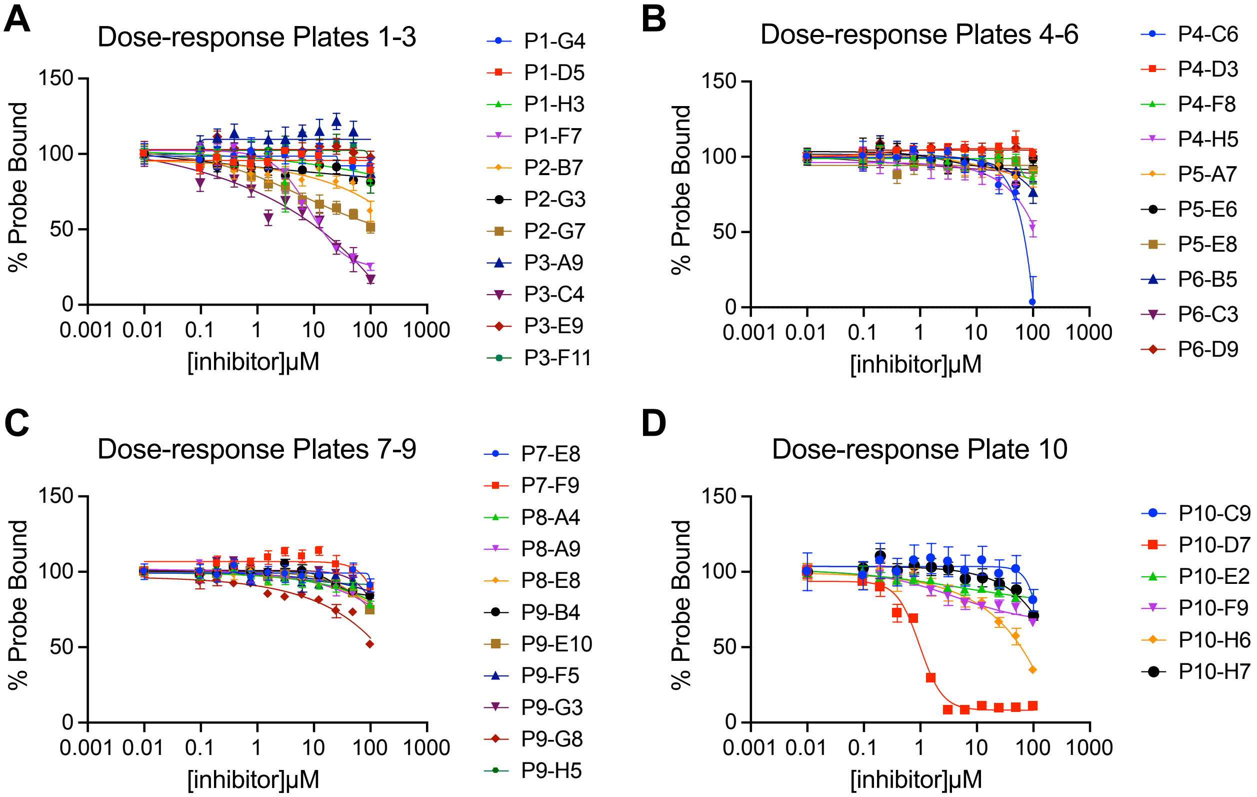

Fig. S1. Dose-response testing of hits from initial single-dose screen of FDA-approved molecules. Background fluorescence polarization (in the absence of PqsE) values were subtracted and measurements were normalized with polarization values at 0 μM competitor compound equal to 100% Probe Bound. Compounds that were re-tested in a dilution series were any initial hits that decreased fluorescence polarization by at least 25% compared to the DMSO control (28 hits) plus some select molecules that did not decrease fluorescence polarization. Notably, one molecule, (S)-Carbidopa, appeared to have significant dose-dependent inhibition, but upon repeated assays, was not a PqsE-binder (panel a, P2-G7). Values are plotted as the average of three technical replicates with error bars representing standard deviation. Inhibition curves were fit to the data in the Prism 9 software.

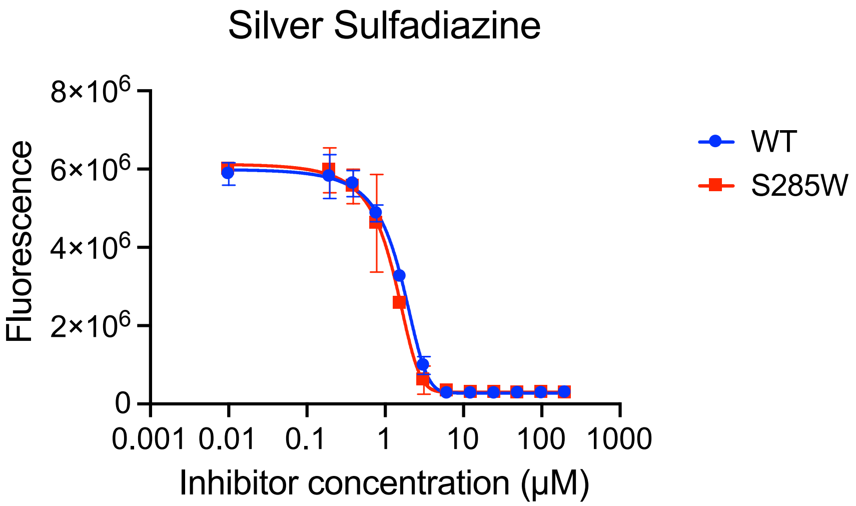

Fig S2: Assessment of Silver Sulfadiazine binding mode. Silver Sulfadiazine was tested for inhibition of the ability of both PqsE(WT) and PqsE(S285W) to hydrolyze the MU-butyrate ester substrate. IC_{50} values against both the WT and variant protein with a partially blocked active site were nearly identical, suggesting that the binding mode of Silver Sulfadiazine does not rely on the active site serine. Values plotted are the average of technical triplicates with error bars representing standard deviation. The data shown are raw, unnormalized fluorescence values.

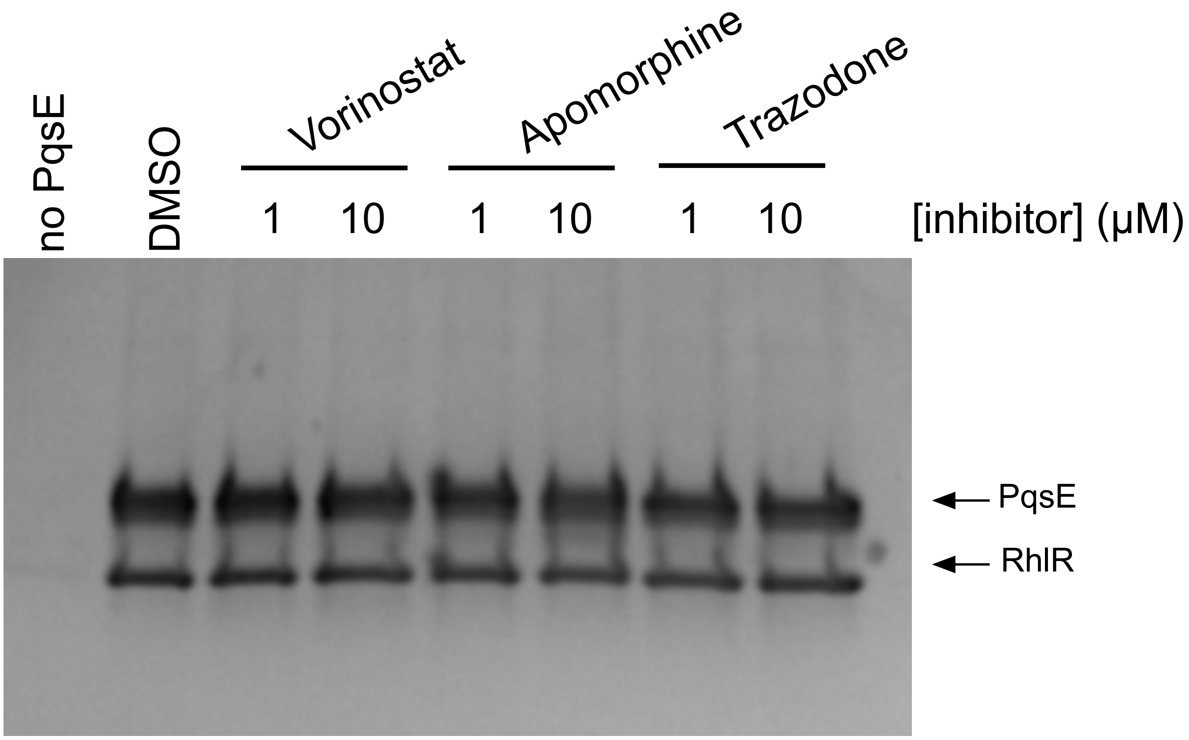

Fig S3: Effect of screening hits on in vitro PqsE-RhlR complex assembly. Purified PqsE with a 6xHis tag was incubated at 1 µM with the compounds Vorinostat, Apomorphine, and Trazodone at the specified concentrations. The PqsE:inhibitor complexes were subsequently incubated with lysate containing RhlR-mBTL and Nickel-coated resin. The resin was washed, proteins were eluted from the resin, and the eluates were subjected to SDS-PAGE. In the left-most lane, no PqsE was added and the RhlR-containing lysate was incubated with the Nickel resin to determine any non-specific binding. PqsE is ~34 kDa and RhlR is ~28 kDa, and the bands corresponding to each protein are labeled to the right of the gel image.

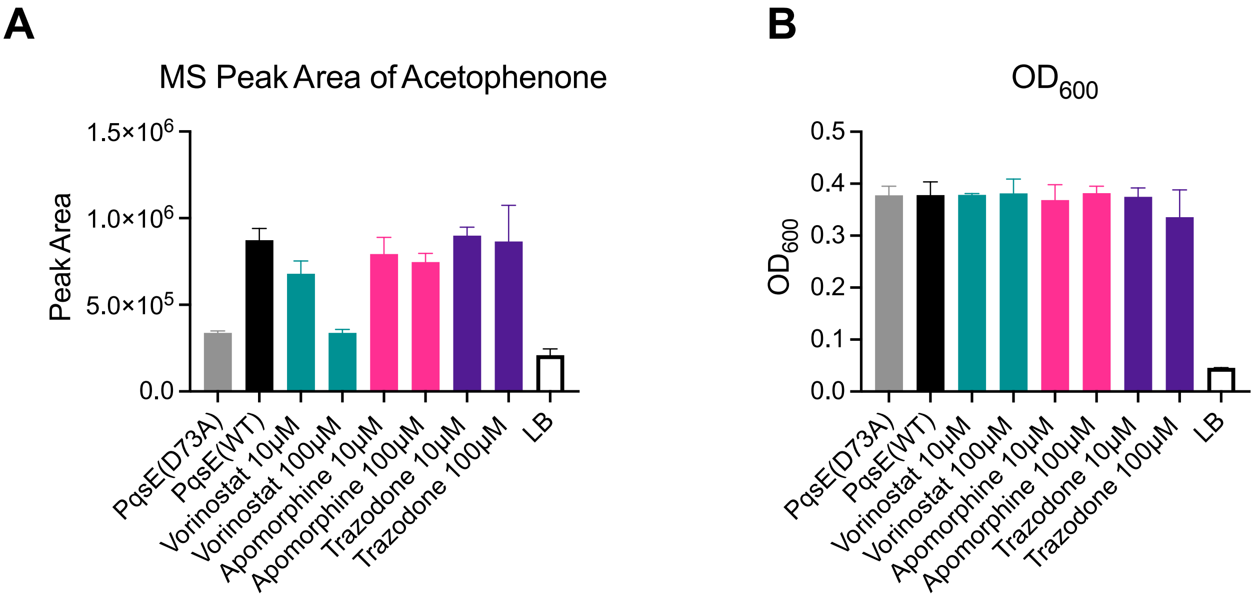

Fig S4: Cellular PqsE enzyme activity assay. A) MS peak area measured for acetophenone in culture supernatants. In all cases, including the PqsE(D73A) and PqsE(WT) controls, cultures were treated with 1% DMSO. Tubes of blank LB were incubated along with treated cultures to establish baseline measurements, which were subtracted from the data set for Vorinostat presented in Figure 5. B) The OD_{600} was measured for each suspended culture at the end of the 8 hr growth period in the presence of the tested inhibitors. MS peak areas were normalized to OD_{600} for the data presented in Figure 5. All data shown are the average of three biological replicates and error bars represent standard deviation.

Supplementary Tables

Strain	Description	Reference
UCBPP-PA14	PA14 *P. aeruginosa* Wildtype	Laboratory stock
SM776	*E. coli* BL21 (DE3) pET28b-6xHis-pqsE(WT)	36
IT55	*E. coli* BL21 (DE3) pET28b-6xHis-pqsE(S285W)	21
IT106	PA14 *pqsE(D73A)*	23
IT245	PA14 Δ*rhlI PazeB-luxCDABE*	This study
BT034	PA14 Δ*rhlI*Δ*pqsE PazeB-luxCDABE*	This study

Table S1. Strains used in this study.

Materials and Methods

Strains, Media, and Chemicals

All experiments with *P. aeruginosa* used the UCBPP-PA14 strain as the parent strain (referred to as PA14). A list of all strains used in this study and their origin is included in **Table S1**. Unless otherwise stated, all cultures were grown in Luria-Bertani (LB) broth (Difco). PqsE was recombinantly expressed and purified as described previously [1,2]. The FDA-approved small molecule library was purchased from Enzo Life Sciences (BML-2843-0100, V.2.0) and screening hits were purchased individually from Enzo for follow-up testing.

Fluorescence Polarization Screen

Methods were derived from Taylor *et. al* 2021[1]. Briefly, PqsE was diluted with assay buffer (50 mM Tricine, 0.01 % Triton X-100, pH 8.5) and added to the wells of an opaque 384-well plate at a final concentration of 2.0 µM. Each well had a final volume of 20 µL with the diluted protein accounting for 10 µL. Screen molecules diluted in DMSO were added to the wells at a final concentration of 250 µM and accounting for 0.5 µL of total well volume (2.5% DMSO final). The plate was incubated at room temperature for ~10 minutes to allow protein-inhibitor complexes to form. The fluorescent probe, BB562, was diluted in assay buffer and added to the wells at a final concentration of 2.0 µM, accounting for 9.5 µL of the total well volume. The plate was then incubated at room temperature for 30 minutes before reading fluorescence polarization. Fluorescence polarization was measured in a Molecular Devices iD5 plate-reader with excitation and emission wavelengths of 485 and 530 nm, respectively. The Prism 9 software was used to generate all graphs and calculate EC_{50} values. In the dose-response FP assay, the inhibitor molecules underwent a two-fold dilution series before being added to the well plate. The top concentration in these assays was 125 µM.

In vitro *Enzyme Assay*

To measure the enzyme activity of PqsE in the presence of an inhibitor, the general Esterase procedure was derived from Taylor et al. 2021[1]. Briefly, purified PqsE, diluted in assay buffer (50 mM Tricine, 0.01% Triton X-100, pH 8.5), was added to an opaque 384-well plate at a final concentration of 125 nM. The potential inhibitory molecules were diluted in DMSO and underwent a 2-fold dilution series to show the dose-dependent nature of their inhibition of enzyme function. Inhibitory molecules were added to the 384-well plate at a top concentration of 100 µM. The plate was incubated at RT for ~5 minutes. MU-butyrate in assay buffer was added to the plate at a final concentration of 2 µM, with a final volume per well of 20 µL. The plate was incubated at RT for 20 min and then placed in a Molecular Devices iD5 plate-reader, where fluorescence of released 4-methylumbelliferone was measured (excitation: 360 nm, emission: 450 nm). To account for inhibitor fluorescence, control wells containing 2 µM MU-butyrate and the inhibitor dilution series were added to the wells in the absence of PqsE, still in a final volume of 20 µL per well. The Prism 9 software was used to generate inhibition curves and determine IC_{50} values. The same procedure was used for esterase assays involving PqsE(S285W).

Dual Pyocyanin/azeB-lux Assay

The pyocyanin assay procedure was derived from Taylor et al. 2021[1]. Briefly, UCBPP PA14 strains of *P. aeruginosa* expressing *pqsE(WT)* or Δ*pqsE* with a chromosomally encoded *azeB-luxCDABE* fusion[3] were grown overnight in LB media at 37 ˚C with shaking. The next morning, the cultures were diluted 1,000x into 2 mL fresh LB, treated with various inhibitor concentrations (1% DMSO), and subsequently grown at 37 ˚C with shaking. After 18 hours of growth, 1 mL of culture was pelleted by centrifugation at 14,000 rpm for 3 min. The supernatants were then collected, and the OD_{695} was measured in a UV/Vis spectrophotometer. The cell pellets were then resuspended in 1 mL PBS. The resuspended cells were loaded into the wells of a white clear-bottomed 96-well plate with 150 µL per well. The cell density (OD_{600}) and luminescence were then measured in a Molecular Devices iD5 plate-reader. Pyocyanin production is reported as OD_{695} normalized to OD_{600} of the resuspended pellet and activation of *azeB-luxCDABE* is reported as luminescence/OD_{600}.

Cellular Enzyme Assay

P. aeruginosa PA14 strains expressing *pqsE(WT)* or *pqsE(D73A)* were grown overnight in LB at 37 ˚C with shaking. The following morning, the cultures were diluted 100x into fresh LB treated with either DMSO or the test compounds in DMSO at the specified concentrations (1% DMSO final). The cultures were prepared in 6 mL total volume in 14 mL polypropylene round bottom tubes and grown at 37 ˚C with shaking at 200 rpm for 8 hr. Each condition was tested in triplicate, including three uninoculated LB blank samples with 1% DMSO. After 8 hr of growth in the presence of the test compounds, the cultures were removed from the incubator and 100 µL from each culture was transferred to a well of a clear bottom 96 well plate in order to measure cell density (OD_{600}) in a Molecular Devices iD5 plate-reader. A low intensity orbital shake was completed prior to measurement to resuspend any settled cells. The remaining cultures were then pelleted by centrifugation at 4 ˚C and 4000 rpm for 10 min. The supernatants were then filter sterilized through a 0.22 µm PES membrane into 20 mL headspace vials and then sealed with a PTFE/silicon cap. Immediately upon completing sample preparation, the headspace vials were transferred to a LECO Pegasus BTX 4D GC×GC-TOFMS instrument equipped with an LPAL autosampler for automated solid-phase microextraction (SPME) arrow headspace sampling and direct thermal desorption at the GC inlet. In the loading rack of the instrument, samples were at room temperature. Prior to injection, each headspace vial was agitated for 2 min at 35 ˚C at 250 rpm. The full details of data collection and instrumental analysis are described below.

SPME Arrow Sampling

Solid phase microextraction (SPME) arrow extraction was conducted with a 1.50 mm wide sleeve divinylbenzene/carbon wide range/polydimethylsiloxane (DVB/C-WR/PDMS) fiber (Restek Corporation, Bellefonte, PA, USA). Sampling was done on 20 mL headspace vials (Restek Corporation) containing 5 mL of supernatant. The fiber was chosen due to the range of analytes this sorbent can collect from headspace samples of a biological nature. Sample extraction and injection was performed using a LECO L-PAL3 Autosampler (LECO Corporation, St Joseph, MI, USA). After preparation, samples were transferred to the autosampler tray of the instrument. Sample incubation was performed

for 2 min at 35 °C at 250 rpm. Sample agitation occurred at intervals of 5 s on followed by 2 s off. Sample extraction was performed for 5 min at 50 °C at 1000 rpm. The needle penetration depth was 40 mm into the sample vial and the penetration speed was 20 mm/s. Injection was performed to a depth of 40 mm at 10 mm/s with a desorb time of 2 min.

Prior to the sampling sequence, the SPME arrow fiber was conditioned at 270 °C for 40 min before the sequence and confirmed to be blank with a fiber blank injection. The SPME arrow fiber was reconditioned for 5 min prior to individual sample injection and for 2 min after injection at 270 °C. This reconditioning procedure was repeated between every sample, and an empty vial blank was included every nine samples.

GC×GC-TOFMS Method

The instrument used for analysis of *P. aeruginosa* supernatants was a Pegasus BTX GC×GC with a Paradigm Shift™ reverse fill-flush (RFF) flow modulator and dual channel detection using a flame ionization detector (FID) and a time-of-flight mass analyzer (LECO Corporation). The carrier gas was ultra-high purity helium (Airgas, Radnor, PA, USA) at a flow rate of 0.5 mL/min. The first-dimension column (^{1}D) was an Rxi-5MS column (20 m × 0.18 mm ID × 0.18 μm d_f, Restek Corporation). The second-dimension column (^{2}D) was an Rxi-17Sil MS (3.7 m × 0.25 mm ID × 0.25 μm d_f, Restek Corporation). The ^{1}D flow rate was 0.5 mL/min and the ^{2}D flow rate was 30 mL/min. The sample loop dimensions were 0.17 m × 0.53 mm ID resulting in a loop volume of 38 μL.

The modulation period was 4 s and the flush time was 158 ms throughout the duration of the run. This resulted in a flush factor of 1.73. The calculated flow to the TOFMS was 1.03 mL/min and the calculated flow to the FID was 37.18 mL/min. The inlet was operated in splitless mode for better detection of low-level analytes. The septum purge flow was 3 mL/min and the inlet purge time was 30 s with a purge flow of 20 mL/min and a total inlet flow of 50.5 mL/min.

The inlet temperature was 250 °C for the entire duration of the run. The initial temperature for the GC oven was 40 °C which was held for 2 min, then the oven was ramped at 5 °C/min until a target temperature of 230 °C was reached, with a final hold of 2 min, resulting in a runtime of 42 min. The transfer line was held at 345 °C and the ion source temperature was 300 °C. The TOFMS operated via electron impact (EI) ionization resulting in an acquisition rate of 100 scans/s and an extraction frequency of 30 kHz for the mass range of 30 – 550 *m/z*. An acquisition delay of 300 s was applied to mitigate solvent effects in the early part of the chromatographic run from saturating the MS signal.

The FID was set to 345 °C and operated at 100 Hz. The flow rate for hydrogen (ultra-high purity, Airgas) fuel was 45 mL/min. The flow rate for air (ultra zero purity, Airgas) was 450 mL/min. The flow rate for nitrogen (ultra-high purity, Airgas) makeup gas was 45 mL/min. The FID detector also had an acquisition delay of 300 Hz.

Data acquisition was performed for both detectors using LECO ChromaTOF software V5.59.02 with data processing V1.2.0.6 (LECO Corporation). GC×GC-TOFMS data was exported as .SMP files to a workstation computer with the same ChromaTOF processing software.

Data Processing

Samples were exported as .SMP files after the chromatographic run to a Network Attached Storage (NAS) system. Files were downloaded from the NAS to an offline data workstation and imported into a ChromaTOF database V5.59.02 with data processor V1.2.0.6 (LECO Corporation). This was done so that files could be then analyzed in ChromaTOF Tile® v1.3.50.0 (LECO Corporation). ChromaTOF Tile reviews raw GC×GC data to identify differences between samples or groups of samples based on class averages. A Fisher ratio test was performed on *pqsE(D73A)* and WT samples to identify molecules that showed significant differences in abundance between catalytically dead and active PqsE and thus could be effective in determining inhibitor effectiveness.

The parameters used for the statistical tests were a ^{1}D tile size of 3 and a ^{2}D tile size of 41 (auto-calculated in the software based on the height and width of a peak in ChromaTOF), ^{1}D retention shift of 0 and a ^{2}D retention shift of 0.01, a S/N threshold of 75, a total of 1 sample must exceed S/N threshold, 1 mass F-ratio to average, a minimum of 3 masses per tile, a minimum mass of 35 *m/*z, and a maximum mass of 550 *m/z*. A Fisher ratio threshold of 20 was applied.

After processing the data, a list of "hits" was generated which included the F-ratio value alongside the mean retention times in the first and second dimension, the quant mass, and a heatmap of relative amounts in each sample class average for each hit. The only "hit" reported was that of acetophenone, with a Fisher ration of 221.10. This hit was accepted based on comparison of its retention time and quant mass in ChromaTOF.

Following the discovery of a molecule effective for determining catalytic inhibition using the TOFMS signal, MS peaks for acetophenone were manually assigned in ChromaTOF to all samples to provide peak areas. MS peak areas for each sample were then transferred into Excel for preliminary analysis, normalization, and baseline subtraction. Lastly, Prism 9 software was used to generate all resulting graphs.

References

(1) Taylor, I. R.; Paczkowski, J. E.; Jeffrey, P. D.; Henke, B. R.; Smith, C. D.; Bassler, B. L. Inhibitor Mimetic Mutations in the Pseudomonas Aeruginosa PqsE Enzyme Reveal a Protein–Protein Interaction with the Quorum-Sensing Receptor RhlR That Is Vital for Virulence Factor Production. *ACS Chem Biol* **2021**, *16* (4), 740–752. https://doi.org/10.1021/acschembio.1c00049.
(2) Mukherjee, S.; Moustafa, D. A.; Stergioula, V.; Smith, C. D.; Goldberg, J. B.; Bassler, B. L. The PqsE and RhlR Proteins Are an Autoinducer Synthase–Receptor Pair That Control Virulence and Biofilm Development in Pseudomonas Aeruginosa. *PNAS* **2018**, 201814023. https://doi.org/10.1073/pnas.1814023115.
(3) Tchadi, B. V.; Derringer, J. J.; Detweiler, A. K.; Taylor, I. R. PqsE Adapts the Activity of the Pseudomonas Aeruginosa Quorum-Sensing Transcription Factor RhlR to Both Autoinducer Concentration and Promoter Sequence Identity. *J Bacteriol* **2025**, e0051624. https://doi.org/10.1128/jb.00516-24.
